# Orthopedic Surgery Matched Applicants Are Publishing More: A Bibliometric Analysis of Research Output

**DOI:** 10.7759/cureus.56210

**Published:** 2024-03-15

**Authors:** Anas M Abbas, Jian H Li, Aadi Pandya, Victoria Wang, Bongseok Jung, Alexandra C Echevarria, Araf M Abbas, Robert E Carrier, Brandon Cemenski, Rohit B Verma, Stephen A Albanese, Randy M Cohn

**Affiliations:** 1 Orthopaedic Surgery, Upstate University Hospital, Syracuse, USA; 2 Orthopaedic Surgery, Northwell Health, New Hyde Park, USA

**Keywords:** residency, research, orthopedic surgery, national resident matching program, medical student, medical education

## Abstract

Introduction

This study analyzed the number of peer-reviewed publications submitted by matriculants prior to applying for the orthopedic surgery residency. The graduating residency classes of 2023 and 2027 were included in the study to understand the trend of publications, to inform aspiring orthopedic surgeons.

Methods

The top, middle, and bottom 10 orthopedic surgery residency programs were identified on the Doximity online website. Matriculants were searched on PubMed and Google Scholar for publication contributions. Variables including number of publications, orthopedic publications, first-author authorship, and H-index were analyzed. A logistic regression model was created, and a t-test was conducted to statistically compare the 2027 and 2023 graduating classes.

Results

Matriculants of the 2023 match had higher numbers of publications, orthopedic surgery-specific publications, first authorships, and h-indices than the matriculants of the 2018 match.

Conclusion

The average number of publications has been observed to increase over four years, indicating an increase in competition to match into orthopedic surgery residency. Publishing in higher numbers may be a good indicator of an applicant’s success in not only matching but also matching into a higher-tier program.

## Introduction

Orthopedic surgery residency is one of the most difficult specialties to match into. Despite the expansion of orthopedic surgery residency programs and available positions, there has been a significant increase in applicants failing to match into orthopedic surgery residency [1]. In 2022, only 60% of medical students successfully matched based on data from the National Resident Matching Program (NRMP) [1]. In 2017, the Accreditation Council for Graduate Medical Education (ACGME) reported a total of 165 orthopedic surgery residency programs offering a total of 727 positions, while the American Osteopathic Association reported a total of 41 programs with 121 positions [2,3]. Following the movement toward a single accreditation of residency programs in 2020, the total number of orthopedic residency programs in 2023 grew to 209 programs with 899 available positions [4]. Meanwhile, the number of applicants increased from 1,013 to 1,470 in this five-year span [5].

Additionally, the United States Medical Licensing Examination (USMLE) Step 1 exam, which was previously relied upon in analyzing applicants, has moved to a pass/fail (P/F) scoring system. Results from the 2022 NRMP revealed that up to 79% of orthopedic surgery program directors (PDs) had a target Step 1 score used to screen applicants to decrease the number of applications to sort through [6]. Step 1 scores were seen as the most important component of applications [6]. With the transition to a pass/fail grading system for Step 1, PDs will have to resort to other aspects of the application when determining the best-fit candidates. As a result, research and publications have gained increasing importance in the evaluation of applicants.

The NRMP Charting Outcomes in the Match includes a self-reported survey completed by medical students during the application process. Research productivity in the form of publications is one quantifiable measure that is reported [7]. In 2022, the average number of research publications reported by United States Medical Doctors was found to be 16.5, 50.82% greater than the national average [7]. This does not include self-reported data from osteopathic medical students or international medical graduates [6,7]. However, this may be a misleading figure as NRMP categorizes abstracts, presentations, and peer-reviewed publications into one data point [7]. In addition, the survey does not distinguish article subtypes (case reports, systematic reviews) or order of authorship [7]. Previous bibliometric analyses of research productivity in determining a successful orthopedic surgery match were conducted prior to the elimination of numeric Step 1 scores [8-10]. As a result, there was a need for accurate representation of research output to properly inform prospective students and mentors. We believed that the regression analysis used in this study provided novel and important information required to more accurately depict the correlation between research output and match results. The logistic regression model has yet to be used in recent bibliometric analyses but is a useful statistical tool to further delve into the descriptive statistics used in previous studies [10].

The purpose of this study was to accurately represent the number of peer-reviewed publications from matriculants prior to submitting their applications into orthopedic surgery residency programs in the US in recent years. Furthermore, we distinguished the author’s involvement in research through the order of authorship, publication in an orthopedics-specific journal, and H-index score. We predicted that the postgraduate year 1 (PGY-1) in the class of 2027 would have a stronger research output than PGY-1 in the class of 2023 before applying for their respective match cycles.

This article was previously presented as a meeting abstract at the ORS 2024 Annual Meeting on February 3, 2024.

## Materials and methods

Search method

Orthopedic surgery residency programs were identified using the Doximity online website. The programs were identified using the website’s residency navigator search tool, which categorized the nation’s orthopedic residencies based on reputation. The residency data displayed on the website is derived from the ACGME database and annual residency navigator surveys coordinated by Doximity. A total of 208 programs were identified. Military programs were excluded from this study, leaving 200 programs in the rank list. The top, middle, and bottom 10 programs were identified from the Doximity website. The first 10 programs on the list were included in the top-tier list. The middle-tier 10 programs were chosen consecutively from the middle of the list starting at program number 105 and including five programs each from above and below that program given that the inclusion criteria were met. The bottom 10 programs were chosen from the end of the list. The individual residency programs were then researched; those programs that had their list of residents updated as of July 20, 2023, were included in the study. Programs that failed to list their current residents were excluded from this study (n=2). For each program, the lists of residents from the classes of 2023 and 2027 were obtained. A literature search was performed for each individual PGY-1 resident to determine their history of work published by September 1 on the year of their application to residency; for residents in the class of 2023, the cutoff date was 09/01/2017, and for residents in the class of 2027, the cutoff date was 09/01/2021.

Resident data were collected by searching their first and last name on PubMed and Google Scholar. The last literature search was performed on December 22, 2023. The title, abstract, and manuscript were analyzed, and the following published articles met the inclusion criteria for peer-reviewed publications: videos, technical notes, correspondence, lectures, letters to editors, case reports and case series, quality improvement, observational studies (case-control, cohort, retrospective, prospective, survey), literature reviews (narrative, systematic, meta-analysis), and original articles (basic science, randomized controlled trial, cadaveric). Errata and abstracts were excluded from publication counts. The authors of this study looked at (1) other publications from the same individual, (2) coauthors, (3) time of publication, and (4) cross-referring author names and affiliations (medical school attended, undergraduate program attended, previous work history). In cases where author names had been changed or varied, the residents’ names were again confirmed by their institutional affiliation, names listed on the residency page, and their history of publication. If the above steps were inconclusive, the residents’ professional profiles on Doximity, ResearchGate, or LinkedIn were reviewed for clarity. If there were any disagreements among the authors, the residents were contacted via social media, or another search was performed using an alternate database for confirmation. The data collected were transferred to Microsoft Excel (Seattle, WA), which included the names of the residents, the digital object identifier associated with each publication, author contributions (first author), orthopedic journal publications, and the H-index. The H-index was obtained by using the online website Resurchify.

Statistical analysis

A logistic regression model was developed to analyze the effect of orthopedic residents’ characteristics on their tier of the residency program. Orthopedic surgery residents’ characteristics included the number of first authorships, the total number of publications, and their H-index average. A t-test was used to compare differences in the number of total publications, orthopedic-specific publications, first authorship, and average H-index scores between similar tiers of programs in 2023 and 2027. Residency program tiers were categorized into tertiles with top, middle, and bottom programs. All analyses were completed in R (version 4.3.2; R Development Core Team, Vienna, Austria). A focus on research publications and their substantiality was selected due to their importance in the training of physicians. According to ACGME, research activities are “intrinsic to the discipline are scientific knowledge, the scientific method of problem-solving, evidence-based decision-making, [and] a commitment to lifelong learning” [11]. PDs appear to mirror this sentiment, with most surveyed PDs considering research when evaluating applicants [12]. This dedication to research during residency training was mirrored in first-year 2021-2022 orthopedic surgery residents, with an average of 16.5 abstracts, presentations, and publications. Lastly, several other studies have evaluated the importance of research in the evaluation of applicants for residency [8-10].

## Results

Overall, the class of 2027 orthopedic surgery residents had a higher average number of total publications (4.12 vs. 1.31, p-value < 0.01), a higher average number of orthopedic-specific publications (2.55 vs. 0.61, p-value < 0.01), a higher average number of first authorships (1.11 vs. 0.35, p-value < 0.01), and a higher average H-index score (78.66 vs. 56.39, p-value < 0.05) than the class of 2023 orthopedic surgery residents.

**Figure 1:**
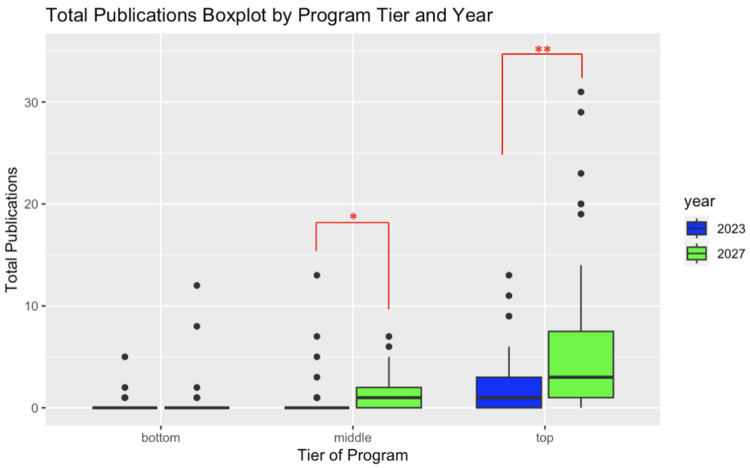
Comparative box plot of the total number of publications between the classes of 2023 and 2027 broken down by program tiers. *: p < 0.05; **: p < 0.01

When examining the total number of publications, the top-tier class of 2027 orthopedic surgery residents had a higher number of total publications (6.91 vs. 2.02, p-value < 0.01) than the top-tier class of 2023 orthopedic surgery residents. Among the middle-tier orthopedic surgery programs, the class of 2027 orthopedic surgery residents had a higher number of total publications (1.42 vs. 0.65, p-value = 0.044). There was no statistically significant difference between the number of total publications among bottom-tier programs (Figure 1).

**Figure 2:**
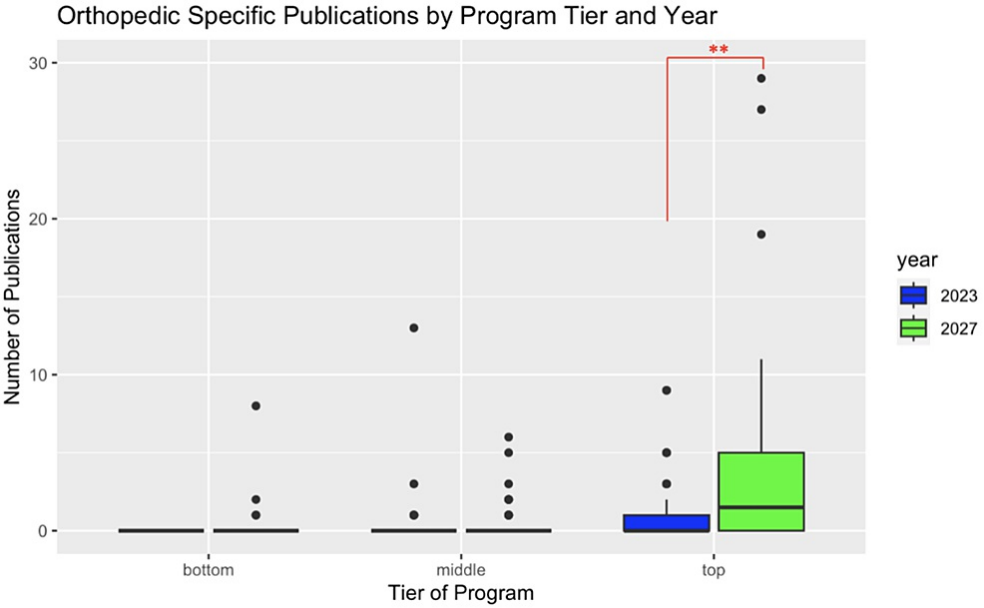
Comparative box plot of orthopedic-specific publications between the classes of 2023 and 2027 broken down by program tiers. **: p < 0.01

When examining the total number of orthopedic-specific publications, the top-tier class of 2027 orthopedic surgery residents had a higher number of publications (4.55 vs. 0.95, p-value < 0.01) than the top-tier class of 2023 orthopedic surgery residents. There was no statistically significant difference between the number of orthopedic-specific publications among the middle- and bottom-tier programs (Figure 2).

**Figure 3:**
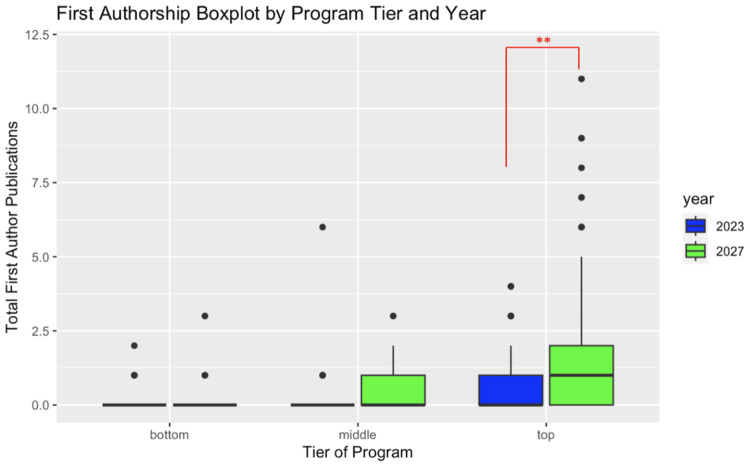
Comparative box plot of first authorship publications between the classes of 2023 and 2027 broken down by program tiers. **: p < 0.01

Within the top-tier orthopedic surgery programs, the class of 2027 orthopedic surgery residents had a higher number of first authorships (1.91 vs. 0.53, p-value < 0.01) than the class of 2023 orthopedic surgery residents. There was no statistically significant difference in the number of first authorships between the classes of 2027 and 2023 orthopedic surgery residents in the middle- and bottom-tier programs (Figure 3).

**Figure 4:**
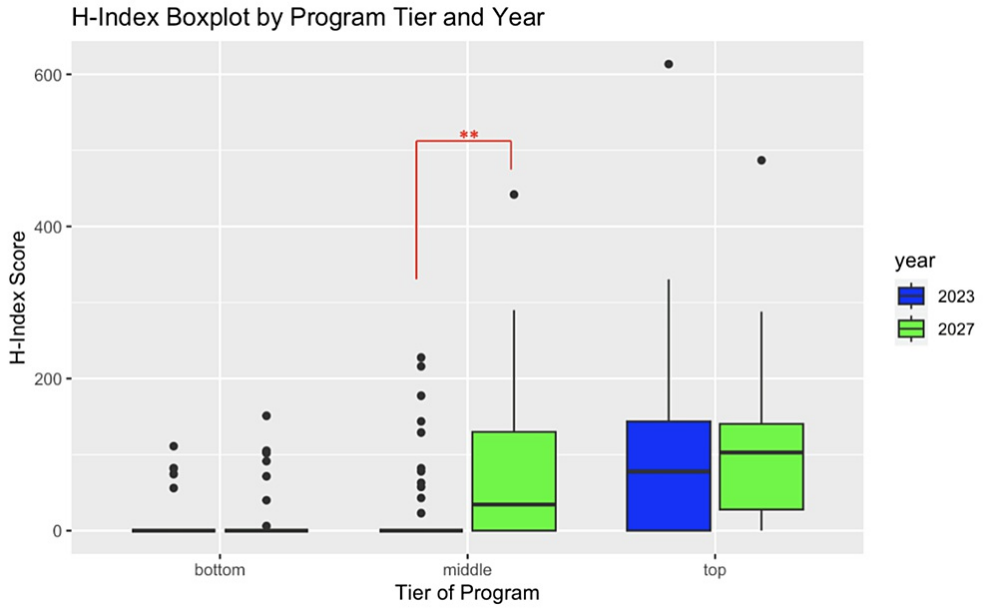
Comparative box plot of the publication H-index between the classes of 2023 and 2027 broken down by program tiers. **: p < 0.01

Among the middle-tier orthopedic surgery programs, the class of 2027 orthopedic surgery residents had a higher average H-index score (77.72 vs. 23.85, p-value < 0.01) than the classes of 2027 and 2023 orthopedic surgery residents. There was no statistically significant difference in the average H-index scores between the classes of 2027 and 2023 orthopedic surgery residents in the top- and bottom-tier programs (Figure 4).

Among the analyzed orthopedic surgery residents in the class of 2027, the number of total publications was a significant positive predictor of which tier of the orthopedic surgery program that resident matched into (p < 0.01). For every additional publication, the odds that a resident moved from a lower tier to a higher tier of the orthopedic residency program increased by 40.9%. The number of first authorships and average H-index scores were not significant predictors of program tier among the class of 2027 orthopedic surgery residents.

Among the class of 2023 orthopedic surgery residents, the average H-index score was a significant positive predictor of the tier of the orthopedic surgery program (p-value < 0.01). For every increase in the average H-index score, the odds that an orthopedic surgery resident went from a lower to higher tier orthopedic surgery residency increased by 1.19%. The number of both total and first authorships were not significant predictors of the program tier among the class of 2023 orthopedic surgery residents.

## Discussion

Previous studies have defined the academic portfolio of successfully matched orthopedic surgery residency applicants [8,9]. However, these studies included applicants who have successfully matched up until the 2016-2017 match cycle (class of 2022), prior to the elimination of Step 1 scores [8,9]. One bibliometric analysis of research productivity within successfully matched applicants included students from the 2013 to 2017 match cohort [8,9]. In 2017, only matriculants in the highest-tiered programs differed significantly in total publications, H-index, and citations [8]. Matriculants with at least one publication matched at a higher-ranked program than matriculants with zero publications, but there was no significance found in matching higher-ranked programs between those with one publication and greater than one publication [8]. Another study investigating the academic portfolios of successfully matched orthopedic applicants evaluated 3,199 applicants from 2013 to 2017 [9]. The median H-index was 0, the median total publication count was one, and 40% of matched applicants did not have a publication at the time of applying [9]. The five-year trend found an increase in the H-index and median total publication count and a decrease in the proportion of applicants holding zero publications prior to applying to the match [9]. Interestingly, the number of first-authored publications had decreased over this time frame [9]. From 2017 to 2022, the NRMP survey reported an increase in the mean number of research products (8.2-16.5) [5,7]. There was a recent bibliometric analysis study on matriculants of the 2020 match (class of 2025 residents) [10]. This study used descriptive statistics to find an average of 3.8 publications per matriculant with higher total publication counts and H-index associated with matching into a top 25 program [10]. With prior studies distinguishing peer-reviewed publications from the data presented in the NRMP and the increase in reported research products, there was a need to scrutinize medical student research productivity in more recent years. Our study provided a unique perspective on recent research output with the use of a logistic regression model. Furthermore, we used matriculants of two different match cycles (2018 and 2022) to assess the shift in focus on research.

Our study showed that the trend of robust research portfolios in matched applicants has continued to grow since 2017. Applicants of the 2022 match had a significantly higher total number of publications (4.12 vs. 1.31, p-value < 0.01), orthopedic-specific publications (2.55 vs. 0.61, p-value < 0.01), first-authored publications (1.11 vs. 0.35, p-value < 0.01), and a higher average H-index score (78.66 vs. 56.39, p-value < 0.05) than the orthopedic surgery matriculants of the 2018 match. This trend was also seen between the respective top-tier programs of each class, except for H-index, while the middle-tier programs had an increase in total publications and higher H-index publications. Between the two classes, the bottom-tier programs did not show significant differences in research output. A bibliometric analysis of applicants from the 2013 to 2017 match cycles found that the average publication was one, further showing that the total number of publications for matriculants has continued to increase over the years [9].

In this study, the most significant positive predictor of matching into a higher-tier program was the H-index for successful applicants in the 2018 match, while the most significant positive predictor for successful applicants matching into a higher-tier program in the 2023 match was the total number of publications. This further substantiated our hypothesis that applicants are looking to publish more than ever to gain a competitive edge. While the most important aspect of one’s research portfolio was to publish meaningful literature in the past, publishing in higher numbers with less focus on impactful journal submissions seemed to be the more recent trend.

Bibliometric analysis in other competitive fields that are research-focused has been done to determine the impact of research output on a medical student’s ability to match [13-16]. These studies slightly varied in their methodology and results but have generally shown an increase in medical student research productivity in the past few years with a correlation of higher research output in matriculants at top-tier residency programs [13-16]. For example, in plastic surgery, one study demonstrated that total publications, plastic surgery-related and unrelated publications, first-author publications, impact factor, and H-index were all associated with matching into both a higher-ranked reputation (p < 0.05) [13]. The average number of publications for interns from 2020 and 2021 was 2.43 ± 3.84 [13].

The discrepancy between the NRMP data (mean 16.5 publications) and our findings (mean 4.12 peer-reviewed publications in the class of 2027 residents) have been noted in studies of other research-heavy specialties and could be attributed to several factors: 1) the subjective nature of self-reported surveys; 2) the lack of distinction between abstracts, presentations, and publications in the survey; and 3) student noncompliance in completing the survey [13-16]. One study found that up to 22% of radiation oncology applicants had misrepresented their publications [17]. Given the vague instructions when listing research on electronics residency application services and NRMP, these misrepresentations may not have been intentional but highlight the need for clarity in the application process.

This study was not without limitations. First, Doximity is a residency navigator tool that ranks programs in descending order based on annual surveys completed by current and graduated residents [18]. Subjective reviews (current resident and recent alumni satisfaction data, reputation data) constitute two-thirds of the ranking process with the remaining one-third made of objective reviews (e.g., alumni research output, board examination pass rate), which ultimately lack objective validity [18]. Our top-tier programs included only the first 10 programs found on the ranking list, while the bottom tier included the last 10 programs on the list that met the inclusion criteria. The middle-tier programs were chosen consecutively from the middle of the ranking list. Thus, our findings may not reflect the true results of our outcome measures, with most programs not included in our study. Nonetheless, it is a widely used platform that has a major influence on applicants and has been included in the methodology of previous bibliometric analyses of research productivity [13-16]. Second, only peer-reviewed publications that were published on or before September 1, 2017, for the class of 2023 and September 1, 2021, for the class of 2027 were included in our study. It is possible that applicants with peer-reviewed publications after this date updated their programs before PDs finalized their rank-to-match lists. Rank-to-match submission deadlines are program-dependent, and this uncertainty led us to exclude publications between September 1 and the match day. Third, the impact of publications and matching was assessed independently of other important aspects of an application such as USMLE scores, LORs, and clerkship grades. Although we excluded abstracts and presentations, these research items may play a major role in match probability. Lastly, there were several residents who changed their surnames between their publications. We accounted for this through careful searching of professional platforms and tracked their volunteering, educational, and work history. Despite our diligent efforts to ensure accurate data collection, it is possible that certain publications were missed and excluded from our study. Further research is needed to evaluate the focus on research after the 2024 match where applicants will have matched with a P/F Step 1 score.

## Conclusions

This study found that the research productivity of those applying for the orthopedic surgery match has increased over a four-year span. In recent years, there has been a shift in focus towards publishing at higher rates, regardless of the impact of the research. We hope that future NRMP surveys will distinguish research parameters to benefit both medical students and PDs.

## References

[1] Lubowitz JH, Brand JC, Rossi MJ (2022). The 2022 orthopaedic surgery residency match leaves many qualified candidates unmatched. Arthroscopy.

[2] National Resident Matching Program (2023). Results and Data: 2017 Main Residency Match®. Data.

[3] National Resident Matching Program (2023). Summary of Positions Offered and Filled by Program Type 2017. https://natmatch.com/aoairp/stats/2017prgstats.html.

[4] National Resident Matching Program (2023). Advance Data Tables 2023 Main Residency Match®. https://www.nrmp.org/wp-content/uploads/2023/04/Advance-Data-Tables-2023_FINAL-2.pdf.

[5] National Resident Matching Program (2023). Results and Data: 2021 Main Residency Match®. https://www.nrmp.org/wp-content/uploads/2021/08/MRM-Results_and-Data_2021.pdf.

[6] National Resident Matching Program (2023). Results of the 2022 NRMP Program Director Survey. https://www.nrmp.org/wp-content/uploads/2022/09/PD-Survey-Report-2022_FINALrev.pdf.

[7] National Resident Matching Program (2023). Charting Outcomes in the Match: Senior Students of U.S. Medical Schools, 2022. https://www.nrmp.org/wp-content/uploads/2022/07/Charting-Outcomes-MD-Seniors-2022_Final.pdf.

[8] Toci GR, Elsner JA, Bigelow BF, Bryant BR, LaPorte DM (2021). Medical student research productivity: which variables are associated with matching to a highly ranked orthopaedic residency program?. J Surg Educ.

[9] Ngaage LM, Mb C, Xue S (2021). The orthopaedic match: defining the academic profile of successful candidates. J Am Acad Orthop Surg.

[10] Adeyeri B, Lee T, Beal T, Huang A, Harrington MA (2023). Analysis of research productivity of orthopedic surgery residency applicants. Cureus.

[11] (2023). Accreditation council for graduate medical education. Program requirements for graduate medical education in internal medicine. http://www.acgme.org.

[12] Melendez MM, Xu X, Sexton TR, Shapiro MJ, Mohan EP (2008). The importance of basic science and clinical research as a selection criterion for general surgery residency programs. J Surg Educ.

[13] Mellia JA, Jou C, Rathi S, Perzia BM, Morel A, Azoury SC, Fischer JP (2021). An in-depth analysis of research output in successful integrated plastic surgery match applicants and factors associated with matching at top-ranked programs. J Surg Educ.

[14] Thangamathesvaran L, M Patel N, Siddiqui SH (2019). The otolaryngology match: a bibliometric analysis of 222 first-year residents. Laryngoscope.

[15] Wadhwa H, Shah SS, Shan J (2019). The neurosurgery applicant's "arms race": analysis of medical student publication in the neurosurgery residency match. J Neurosurg.

[16] Huang A, Gunther JR, Lin LL (2022). Analyzing the role of research in the radiation oncology match. Adv Radiat Oncol.

[17] Yang GY, Schoenwetter MF, Wagner TD, Donohue KA, Kuettel MR (2006). Misrepresentation of publications among radiation oncology residency applicants. J Am Coll Radiol.

[18] Feinstein MM, Niforatos JD, Mosteller L, Chelnick D, Raza S, Otteson T (2019). Association of doximity ranking and residency program characteristics across 16 specialty training programs. J Grad Med Educ.

